# Causality in Reversed Time Series: Reversed or Conserved?

**DOI:** 10.3390/e23081067

**Published:** 2021-08-17

**Authors:** Jakub Kořenek, Jaroslav Hlinka

**Affiliations:** 1 Institute of Computer Science, Czech Academy of Sciences, Pod Vodarenskou Vezi 271/2, 182 07 Prague, Czech Republic; korenek@cs.cas.cz; 2 Faculty of Nuclear Sciences and Physical Engineering, Czech Technical University, Brehova 7, 115 19 Prague, Czech Republic; 3 National Institute of Mental Health, Topolova 748, 250 67 Klecany, Czech Republic

**Keywords:** causality, time reversal, temporal symmetry, reversed time series, vector autoregressive process, random networks, brain network, climate network

## Abstract

The inference of causal relations between observable phenomena is paramount across scientific disciplines; however, the means for such enterprise without experimental manipulation are limited. A commonly applied principle is that of the cause preceding and predicting the effect, taking into account other circumstances. Intuitively, when the temporal order of events is reverted, one would expect the cause and effect to apparently switch roles. This was previously demonstrated in bivariate linear systems and used in design of improved causal inference scores, while such behaviour in linear systems has been put in contrast with nonlinear chaotic systems where the inferred causal direction appears unchanged under time reversal. The presented work explores the conditions under which the causal reversal happens—either perfectly, approximately, or not at all—using theoretical analysis, low-dimensional examples, and network simulations, focusing on the simplified yet illustrative linear vector autoregressive process of order one. We start with a theoretical analysis that demonstrates that a perfect coupling reversal under time reversal occurs only under very specific conditions, followed up by constructing low-dimensional examples where indeed the dominant causal direction is even conserved rather than reversed. Finally, simulations of random as well as realistically motivated network coupling patterns from brain and climate show that level of coupling reversal and conservation can be well predicted by asymmetry and anormality indices introduced based on the theoretical analysis of the problem. The consequences for causal inference are discussed.

## 1. Introduction

The temporal symmetry of physical processes, as well as the common fundamental lack of it, is among the most fascinating natural phenomena with deep theoretical consequences. Its characterization has been the topic of statistical physics including intriguing fundamental questions concerning quantum processes [1] as well as nonlinear time series analysis methods capturing the nonlinear aspects of brain electrophysiology [2]. Interestingly, it is intricately related to another fundamental concept used by humans to make sense of the processes in their natural environment—that of *causality*, with the notion of cause and effect as generalization of the naive observation that under (seemingly) equivalent circumstances, performing (or not) an action is followed by different event scenarios. While it is generally accepted that to uncover the causal principles ruling a given system one would need to be able to probe the systems responses experimentally [3], a range of methods were proposed that aim for estimation of the (network of) causal interactions just from the observed time series.

One of the prominent ideas behind a wide family of these methods is the principle that causality relates to predictability in that a variable can be considered causal with respect to another, if using the current state of the former improves the prediction of the latter [4]. To avoid false inference due to possible shared information in the past or due to other variables, these other variables are in theory supposed to be also observed and used in both of the compared prediction models; of course in practice there are limitations on available observational data as well as estimability of the models that lead to the numerical methods providing only estimates of causal structure. However, the general definition represents an elegant and natural formalization of the intuitive understanding that the cause should precede the effect and carry some information about it (i.e., be at least statistically, if not deterministically related to the effect).

Notably, the relation between the two fundamental concepts of temporal symmetry and causality has recently become thematized [5,6,7,8,9,10]. One of the interesting conjectures, supported by observations from simulated processes, was that for linear processes, the direction of causal interaction is reversed upon the reversal of the direction of time, while it is conserved for nonlinear or chaotic time series [6,8]. The observation of causal direction reversal upon time-reversal in linear systems was previously noted in multiple studies, and previously used for construction of more robust causality scores [5,9,11], shown to potentially fail under the influence of an unobserved latent variable [8] (which is an important classical example of limitation of Granger causality and other time series analysis methods, which theoretically assume that all relevant variables are observed), and confirmed for Granger causality while not observed for a novel Compression-Complexity Causality measure [10].

In this work, we follow the observations in linear processes in more depth. In particular, we extend the analysis to multivariate systems, i.e., networks. For arbitrary network size, we start by building upon an almost forgotten result of Anděl [12] and derive the necessary and sufficient condition of causal structure reversal under time reversal for autoregressive processes of order 1; in this simplified model it turns out the key property is the normality of the causal interaction matrix, albeit for higher model orders the existence of such simplified conditions remains an open problem. In the previously studied bivariate case, we provide a simple analytic derivation illustrating that the causal structure reversal almost never happens perfectly, but only approximately (apart from two rather trivial cases with particular coupling symmetry), in line and further elucidating the previous reports of observations of approximate albeit not exact causal reversal. We consequently show that the minimal unidirectionally coupled network for which the exact causal structure reversal appears has 3 subsystems, and document this by an example. We finally turn to real-world data and simulation scenarios, studying the extent to which the causal structure reversal appears in linear vector autoregressive order one approximation of a brain and climate network under time reversal. The results show that already the linear approximations of both brain and climate systems show imperfect causal structure reversal under time reversal, while the extent to which this property is broken is closely predicted by deviation of the coupling matrix from normality (rather than from pure symmetry of the matrix) in both realizations of randomly connected networks as well as these more realistic connectivity structure scenarios. Finally, we discuss the relevance of these findings in a wider context, including how it problematizes the use of comparison with time-reversed version of a given process for inference of causal structures.

## 2. Theoretical Analysis

### 2.1. Reversed Autoregressive Process

In 1972, Anděl [12] proved a theorem describing a relation between a vector autoregressive process and its reversed process. First, we define the stationary autoregressive process and its reversed process according to his notation.

**Definition** **1** (Autoregressive process)**.** *Let ${\left\{Y_{t}\right\}}_{t=-\infty }^{+\infty }$ be a series of uncorrelated n-dimensional random vectors such that $E[Y_{t}]=0$ and covariance matrix of vector $Y_{t}$, $\mathrm{Var}[Y_{t}]=I$. Let $A_{0},\ldots ,A_{n}$ be matrices from $R^{n,n}$ such that:*

1. $detA_{0}\neq 0$
2. $A_{n}\neq 0$
3. *the equation $det\left(\sum_{j=0}^{p}A_{j}\lambda^{p-j}\right)=0$ has all the roots smaller than 1 in absolute value*

*and let ${\left\{X_{t}\right\}}_{t=-\infty }^{+\infty }$ be a series defined by the recurrent formula*

(1) $$\begin{array}{c} \sum_{j=0}^{p}A_{j}X_{t-j}=Y_{t}\;\;\;\;-\infty <t<+\infty , \end{array}$$

*or equivalently*

(2) $$\begin{array}{c} X_{t}=-\sum_{j=1}^{p}A_{0}^{-1}A_{j}X_{t-j}+A_{0}^{-1}Y_{t}\;\;\;\;-\infty <t<+\infty . \end{array}$$

*Then $\left\{X_{t}\right\}$ is n-dimensional autoregressive process of order p (which is stationary due to conditions above) with the covariance function $R_{jk}\left(t\right)=E\left[X_{t}^{j}X_{0}^{k}\right].$*

Please note that for an autoregressive process, the set of matrices $A_{i}$ fully describes the causal interactions in the system.

**Definition** **2** (Reversed process)**.** *We say that $\left\{Z_{t}\right\}$ is* reversed *(in time) with respect to an autoregressive process $\left\{X_{t}\right\}$ with covariance function $R_{jk}\left(t\right)$, if $\left\{Z_{t}\right\}$ has the matrix of the covariance functions ${\left(R_{jk}\left(-t\right)\right)}_{j,k=1}^{n}.$*

Anděl [12] proved the following theorem.

**Theorem** **1.** *Let $\left\{Z_{t}\right\}$ be the n-dimensional autoregressive process defined as*

(3) $$\begin{array}{c} Z_{t}=-\sum_{j=1}^{p}B_{0}^{-1}B_{j}Z_{t-j}+B_{0}^{-1}Y_{t}\;\;\;\;-\infty <t<+\infty , \end{array}$$

*The series $\left\{Z_{t}\right\}$ is reversed with respect to $\left\{X_{t}\right\}$ if and only if its autoregressive matrices satisfy following conditions:*

1. $detB_{0}\neq 0$
2. $B_{1}\neq 0$
3. *the equation $det\left(\sum_{j=0}^{p}B_{j}\lambda^{p-j}\right)=0$ has all the roots smaller than 1 in absolute value*

*and*

(4) $$\begin{array}{c} \sum_{k=0}^{p-h}A_{h+k}^{⊤}A_{k}=\sum_{k=0}^{p-h}B_{k}^{⊤}B_{k+h}\;\;\;\;0\leq h\leq p. \end{array}$$

From the theorem, we know that the relation between the original process and the reversed process is given by p+1 Equation (4). For simplicity, in the following we limit the analysis to the simplest but most commonly treated case of a system with delayed interactions with lag of one time step (p=1) and with white noise, i.e., $A_{0}^{-1}=I$ (therefore $X_{t}=-A_{1}X_{t-1}+Y_{t}$). In this case, the causal structure is given by a single matrix $A:=-A_{0}^{-1}A_{1}=-A_{1}$, which we shall further denote as the *coupling* matrix. Please note that non-zero elements of this matrix correspond to pairs of variables between which there is causal connection in the Granger sense, in particular, non-zero value of $A_{ij}$ indicates non-zero input from $X_{j}$ to $X_{i}$, i.e., causal effect in the direction from *j* to *i*.

In this case the set of Equation (4) reduces to the following two matrix equations:

(5) $$\begin{array}{cc} A_{1}^{⊤} & =B_{0}^{⊤}B_{1} \end{array}$$

(6) $$\begin{array}{cc} I+A_{1}^{⊤}A_{1} & =B_{0}^{⊤}B_{0}+B_{1}^{⊤}B_{1}. \end{array}$$

We will derive the conditions under which the causality in the reversed process is reversed to the causality in the original process, i.e., when the coupling matrix of the reversed process $B:=-B_{0}^{-1}B_{1}$ is equal to the transpose of the coupling matrix of the original process A.

Let us assume that $A=B^{⊤}$; then $A_{1}^{⊤}=B_{0}^{-1}B_{1}$, i.e., $B_{1}=B_{0}A_{1}^{⊤}$. By substitution for $B_{1}$ to (6) and (6) we obtain:

(7) $$\begin{array}{cc} A_{1}^{⊤} & =B_{0}^{⊤}B_{0}A_{1}^{⊤} \end{array}$$

(8) $$\begin{array}{cc} I+A_{1}^{⊤}A_{1}=B_{0}^{⊤}B_{0}+ & A_{1}B_{0}^{⊤}B_{0}A_{1}^{⊤}=B_{0}^{⊤}B_{0}+A_{1}A_{1}^{⊤}. \end{array}$$

Further, from Equation (8) we have that $B_{0}^{⊤}B_{0}=I+A_{1}^{⊤}A_{1}-A_{1}A_{1}^{⊤}$. By substituting to Equation (7) we obtain

(9) $$\begin{array}{cc} A_{1}^{⊤} & =\left(I+A_{1}^{⊤}A_{1}-A_{1}A_{1}^{⊤}\right)A_{1}^{⊤}, \end{array}$$

which further simplifies to:

(10) $$\begin{array}{cc} 0 & =\left(A_{1}^{⊤}A_{1}-A_{1}A_{1}^{⊤}\right)A_{1}^{⊤}. \end{array}$$

Equation (10) thus constitutes a *necessary* condition for coupling matrix reversal. Please note that under the reasonably general assumption that A (and therefore $A_{1}^{⊤}$) is regular, the range of the operator $A_{1}^{⊤}$ is the full space $R^{n}$ and thus Equation (10) holds *only* if $0=A_{1}^{⊤}A_{1}-A_{1}A_{1}^{⊤}$, i.e., $A_{1}$ is normal. On the other hand, any normal matrix $A_{1}$ fulfils Equation (10), and together with an arbitrary choice of a unitary matrix $B_{0}$ and $B_{1}=B_{0}A_{1}^{⊤}$ it fulfils the original equations Equations (5) and (6).

Overall, we proved that for VAR(1) process with normal coupling matrix $A_{1}$ and white noise ($A_{0}=I$), the coupling structure in the reversed process is exactly reversed to coupling matrix of the original process. We also proved that for a regular matrix $A_{1},$ observation of the reversed coupling structure in the reversed process implies normality of the coupling matrix $A_{1}.$ Therefore, under the assumption of regularity of $A_{1}$, normality of $A_{1}$ is equivalent to the observation of reversed causality in the reversed process.

A specific subset of normal matrices are symmetric matrices. From the above results concerning normal matrices, we thus learn that for symmetric matrices the causality of time-reversed process is also reversed, which due to its symmetry means that it is *invariant* (conserved) on time reversal of the time series (as the coupling matrix and its transpose are identical). One may ask whether there is a broader family of coupling matrices for which the coupling matrix of the original process and the reversed process is identical. Assuming that $A_{1}=B_{0}^{-1}B_{1},$ then $B_{1}=B_{0}A_{1}$ and from (5) and (6) follows:

(11) $$\begin{array}{cc} A_{1}^{⊤} & =B_{0}^{⊤}B_{0}A_{1} \end{array}$$

(12) $$\begin{array}{cc} I+A_{1}^{⊤}A_{1}=B_{0}^{⊤}B_{0}+ & A_{1}^{⊤}B_{0}^{⊤}B_{0}A_{1}. \end{array}$$

Under assumption of the regularity of $A_{1}$ then follows $B_{0}^{⊤}B_{0}=A_{1}^{⊤}A_{1}^{-1}$ and

(13) $$\begin{array}{c} I+A_{1}^{⊤}A_{1}=A_{1}^{⊤}A_{1}^{-1}+A_{1}^{⊤}A_{1}^{⊤}, \end{array}$$

from which we obtain the condition

(14) $$\begin{array}{c} \left(A_{1}-A_{1}^{-1}\right)={\left(A_{1}-A_{1}^{-1}\right)}^{⊤}. \end{array}$$

In conclusion, we found normality and symmetry of the coupling matrix as sufficient conditions for reversal and conservation of the coupling under time-reversal, albeit in principle these may not be necessary depending on the existence of other eligible solutions for Equations (10) and (14)—we leave the characterization of the full set of solutions for these matrix equations under the additional requirements of Definition 1 and Theorem 1 as an open problem for further work, although based on preliminary analysis we conjecture the solutions might in fact be equivalent with the normality and symmetry condition, respectively.

### 2.2. Bivariate Case

Above, we discussed the conditions for the reversed process to have reversed, or conserved causality for multivariate autoregressive processes (for simplicity limiting the analysis to processes of order 1, with white noise). Let us analyze in more detail the bivariate case, i.e., model of the systems with two elements *X* and *Y*.

The convention:

(15) $$\begin{array}{c} A=-A_{1}=\left(\begin{array}{cc} a & b\\ c & d \end{array}\right), \end{array}$$

leads after substitution to Equation (10) to the set of equations:

(16) $$\begin{array}{cc} -ac^{2}+abc+b^{2}d-bcd & =0,\\ -c^{3}+b^{2}c-cd^{2}+acd+bd^{2}-abd & =0,\\ -b^{3}-a^{2}b+bc^{2}+abd+a^{2}c-acd & =0,\\ -b^{2}d+bcd+ac^{2}-abc & =0, \end{array}$$

which has two types of solutions:

(17) $$\begin{array}{c} A=\left(\begin{array}{cc} a & b\\ b & d \end{array}\right),\;\mathrm{and}\;A=\left(\begin{array}{cc} a & b\\ -b & a \end{array}\right), \end{array}$$

in the sense that all solutions have this form and any matrix of this form is a solution. These are specific cases of either a symmetric matrix (c=b) or a specific case of an off-diagonally antisymmetric matrix (c=−b,d=a).

A particularly interesting case is that of unidirectional coupling, without loss of generality one can focus on the case where *Y* affects *X*, but *X* does not affect *Y*. This model is given by the matrix:

(18) $$\begin{array}{c} A=\left(\begin{array}{cc} a & b\\ 0 & d \end{array}\right), \end{array}$$

where b≠0, while c=0.

Please note that this is the canonical model of unidirectional causality in a linear system, that was used to illustrate the conjecture that in a linear system (unlike in nonlinear, in particular chaotic, systems), time-reversal leads to reversal of the direction of causality [6].

Clearly, this is a specific case of the bivariate system considered above, and due to the condition b≠0, it is not among the solutions for Equation (10). Therefore we know that in this model a *perfect* reversal of causal structure with time reversal does not occur, albeit the *main/stronger/dominant*, direction of causality indeed typically reverts, as discussed by Paluš et al. [6] for simple linear systems, while giving counterexamples for other, typically chaotic nonlinear systems. Please note that while Paluš et al. [6] discussed the coupling direction reversal in context of specific causal indices, in particular the conditional mutual information, the notion of comparing strength of causality in one and the other direction between two variables of the system is used in practice, and under some assumptions comparing the strength of some index of causality helps inference on causal direction from observed data [5,11]. However, the use of the comparison of arbitrary causal indices in both directions is not necessarily valid and might in principle provide different and indeed wrong answers. For instance, one can conceive scenarios where stronger coupling coefficient in the dynamical equations provides a weaker statistical significance due to different temporal scales of the dynamics or signal complexity [13]. Additionally, the value of the coupling coefficient can give a different result than of some causality index. We shall get back to the topic of conservation of the dominant direction of causality in the Discussion section, where we shall separately consider the values of the coupling coefficients, and of the Granger causality indices.

Let us study the bivariate system with unidirectional coupling in more detail. As shown earlier, in case that A is regular, perfect causal reversal occurs if and only if A is normal. Even without relying on the provided solutions of the general conditions (16) one can easily show that there is no normal matrix among regular matrices of type (18), i.e., among those that fulfill both a≠0 and d≠0. In particular, for the triangular coupling matrix A, the condition of normality $AA^{⊤}=A^{⊤}A$ rewrites as:

(19) $$\begin{array}{cc} \left(\begin{array}{cc} a^{2}+b^{2} & bd\\ bd & d^{2} \end{array}\right) & =\left(\begin{array}{cc} a^{2} & ab\\ ab & b^{2}+d^{2} \end{array}\right) \end{array}$$

and therefore $a^{2}+b^{2}=a^{2}$ which is in contradiction with b≠0.

It is also illustrative to consider the singular cases. While perfect causal reversal does not occur (unless in the trivial case of b=0), in one of the cases at least the binary structure of the causal links is reversed (albeit not the exact coefficients). There are only two options such that $-A_{1}$ is singular: a=0 or d=0.

If d=0,

(20) $$\begin{array}{c} A=\left(\begin{array}{cc} a & b\\ 0 & 0 \end{array}\right), \end{array}$$

which leads to (See Appendix A for detailed derivation):

(21) $$\begin{array}{c} B=\left(\begin{array}{cc} a & 0\\ \frac{b\left(1-a^{2}\right)}{1+b^{2}} & 0 \end{array}\right). \end{array}$$

Please note that perfect causal reversal does not happen as $b=\frac{b\left(1-a^{2}\right)}{1+b^{2}}$ only if $a^{2}+b^{2}=0,$ which is in contradiction with the assumption b≠0.

If a=0,

(22) $$\begin{array}{c} A=\left(\begin{array}{cc} 0 & b\\ 0 & d \end{array}\right), \end{array}$$

the reverse process has a coupling matrix

(23) $$\begin{array}{c} B=\frac{1}{1+b^{2}}\left(\begin{array}{cc} b^{2}d & bd^{2}\\ b & d \end{array}\right). \end{array}$$

Here, the reverted process shows *bidirectional* coupling, and perfect causal reversal happens again only in the trivial case of $b=\frac{b}{1+b^{2}}$, i.e., b=0. See Appendix A for detailed derivation.

**Figure 1:**
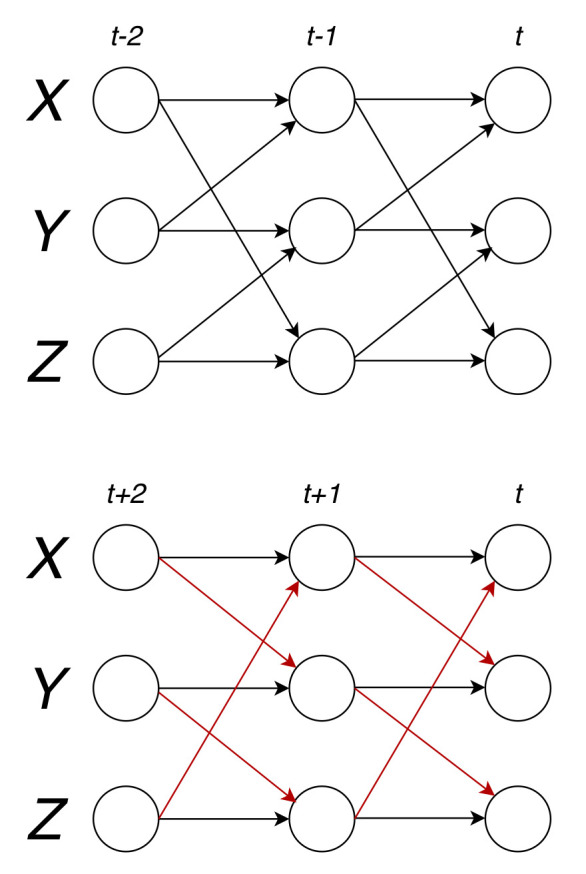
System with normal coupling matrix and its reversed system.

As we showed that perfect causal reversal does not happen for unidirectionally coupled bivariate AR(1) models with white noise, it is a natural question whether it can happen at all in such systems, and in which dimension. The answer is that the smallest dimension in which a perfect causal reversal occurs in a nontrivial matrix is three. Consider the example system with three elements with a normal coupling matrix A the structure of which is depicted in Figure 1.

The binary structure of the coupling matrix of this system is:

(24) $$\begin{array}{c} S=\left(\begin{array}{ccc} 1 & 1 & 0\\ 0 & 1 & 1\\ 1 & 0 & 1 \end{array}\right), \end{array}$$

and by rescaling it by a suitable coefficient $\frac{s}{\lambda_{\max}},$ where $\lambda_{\max}$ is the largest eigenvalue (in absolute value) of the matrix S, and s∈(0,1) is an optional parameter—in this case s=0.8—we assure stationarity of the corresponding AR process. A minimal example of a unidirectionally coupled AR system exhibiting perfect coupling reversal upon time reversal would thus be given by for instance:

(25) $$\begin{array}{c} A=\left(\begin{array}{ccc} 0.4 & 0.4 & 0\\ 0 & 0.4 & 0.4\\ 0.4 & 0 & 0.4 \end{array}\right). \end{array}$$

As the matrix A is normal, the coupling matrix B of the reversed process is exactly the transpose of A:

(26) $$\begin{array}{c} B=\left(\begin{array}{ccc} 0.4 & 0 & 0.4\\ 0.4 & 0.4 & 0\\ 0 & 0.4 & 0.4 \end{array}\right). \end{array}$$

## 3. Numerical Simulations

In the previous section, we discussed the conditions under which the time reversed process has reversed or conserved coupling matrix. In particular, we showed that the sufficient (and under regularity even necessary condition) for the former is the normality of the coupling matrix $A_{1}$, while for the latter (conservation), the condition is given by Equation (14); which is clearly fulfilled for any symmetric matrix $A_{1}$.

In real-world systems, one might expect that the coupling matrix does not fulfill the normality condition, and therefore even in linear auto-regressive systems, the causal structure of the time-reversed process may thus not be exactly reversed. Indeed, already the analysis of the bivariate case showed that strict reversal generally does not occur, and unidirectionally coupled system might show as bidirectionally coupled after time reversal; in fact this is the *generic* case in case the coupling source being autocorrelated due to self-coupling. Therefore it is natural to ask to what extent does the reversed time series causal structure differ from the exactly reversed in example real-world complex systems, and whether this deviation from coupling reversal is somehow related to (and can be reasonably predicted from) the properties of the coupling matrix. In particular, we conjecture that it might be well approximated by the quantitative deviation from the normality condition that would enforce the exact coupling reversal.

### 3.1. Measures Introduction

In the following, we use numerical simulations to show that the level to which the causality is reverted (conserved) on time reversal is indeed well predicted by the deviation of the coupling matrix from normality (symmetry). In other words, that the difference between the matrices A and $B^{⊤}$ strongly depends on the level (a)normality of A, and likewise the difference between A and B depends on the (a)symmetry of A. To this end, we will first formally introduce indices of anormality and asymmetry. Please note that these indices are intended to characterize these particular properties of the matrix itself, rather than provide some information concerning the existence of specific causal links (so they are not to be confused with various causality indices used in causal inference). In particular, the analyses below suggest that the indices of deviation of the coupling matrix from symmetry and normality correlate substantially with the level of causal reversal/conservation, and can thus provide a proxy for whether causal reversal/conservation can be expected in particular (type of) causal structure. Despite their theoretical usefulness for understanding of the causal reversal/conservation behaviour, their practical application might be of course limited by the fact that many real-world systems are not well approximated by a VAR(1) process.

For a square matrix A we define the symmetric part of the matrix $A_{s}=\frac{1}{2}\left(A+A^{⊤}\right)$ and the anti-symmetric part $A_{a}=\frac{1}{2}\left(A-A^{⊤}\right)$. The deviation from symmetry is then defined as $\frac{\left|\right|A_{a}\left|\right|}{\left|\right|A_{s}\left|\right|},$ where ||·|| is some matrix norm. In this approach, for a symmetric matrix the value of the deviation from symmetry is equal to zero and for an antisymmetric it tends to infinity, and that for non-negative matrices it is normalized into the [0,1] interval.

In this paper, we use the Frobenius norm which is for a square matrix $A\in R^{n,n}$ defined as

(27) $$\begin{array}{c} {\left||A|\right|}_{F}={\left(\sum_{i=1}^{n}\sum_{j=1}^{n}{\left|a_{ij}\right|}^{2}\right)}^{1/2}. \end{array}$$

The resulting measure of asymmetry—the deviation from symmetry—is then defined as

(28) $$\begin{array}{c} d_{Sym}\left(A\right)=\frac{||A-A^{⊤}{\left|\right|}_{F}}{||A+A^{⊤}{\left|\right|}_{F}}. \end{array}$$

Based on the same idea, we define a deviation from normality (matrix is normal if $AA^{⊤}=A^{⊤}A$) as

(29) $$\begin{array}{c} d_{Norm}\left(A\right)=\frac{||AA^{⊤}-A^{⊤}A{\left|\right|}_{F}}{||AA^{⊤}+A^{⊤}A{\left|\right|}_{F}}. \end{array}$$

From the analysis outlined in Section 2, we know that a relation $A=B^{⊤}$ holds for normal matrices ($AA^{⊤}=A^{⊤}A$) as well as A=B holds for symmetric matrices ($A=A^{⊤}$). In the following simulation we will show the relation between the deviation of matrix A from symmetry (28) and the difference between matrices A and B. For this purpose we define the normalized difference of these matrices as

(30) $$\begin{array}{c} {\overset{\leftarrow }{d}}_{Sym}\left(A\right)=\frac{||A-{B||}_{F}}{||A+{B||}_{F}}. \end{array}$$

Additionally, we investigate the relation between anormality (29) and the normalized difference between A and $B^{⊤}$, defined as

(31) $$\begin{array}{c} {\overset{\leftarrow }{d}}_{Norm}\left(A\right)=\frac{||A-B^{⊤}{\left|\right|}_{F}}{||A+B^{⊤}{\left|\right|}_{F}}. \end{array}$$

Please note that we consider processes with white noise (zero mean, variance equals to one, that is VAR(1) process in the form

(32) $$\begin{array}{c} X_{t}=AX_{t-1}+Y_{t}, \end{array}$$

with white noise $Y_{t}$.

### 3.2. Simple Causal Structures

In this part we will support theoretical analysis with numerical simulations. According to the expression (32) the time series of length *T* are generated with different choices of matrix A. These time series are then reversed in time, and the coupling matrix is subsequently estimated using the *arfit* function implemented in the MATLAB software.

As a validation example, we use the matrix A introduced in Equation (25). We obtain the following example estimates for time series of length $T\in \{10^{2},10^{4},10^{6}\}$ respectively, showing reasonably fast convergence of the estimates to the theoretically derived matrix B given in Equation (26):

(33) $$\begin{array}{c} B∼\left(\begin{array}{ccc} 0.290 & 0.119 & 0.363\\ 0.212 & 0.397 & 0.005\\ 0.152 & 0.336 & 0.286 \end{array}\right),\left(\begin{array}{ccc} 0.387 & -0.009 & 0.391\\ 0.388 & 0.400 & 0.015\\ -0.012 & 0.410 & 0.401, \end{array}\right),\left(\begin{array}{ccc} 0.400 & 0.002 & 0.400\\ 0.402 & 0.399 & 0.001\\ 0.000 & 0.399 & 0.401 \end{array}\right). \end{array}$$

In the next simulation we explore to what level does the deviation from normality $d_{Norm}\left(A\right)$ determine the imperfection of causal structure reversal ${\overset{\leftarrow }{d}}_{Norm}\left(A\right)$, and similarly how does the deviation from symmetry $d_{Sym}\left(A\right)$ relate to the difference between the causal structure of the forward and time-reversed process ${\overset{\leftarrow }{d}}_{Sym}\left(A\right)$.

For this purpose, we use network topologies defined by a random binary directed graph given by a binary matrix of dimension n×n with value 1 of the diagonal elements (corresponding to loops) and with a total density *D* of nonzero elements. Each such realization is scaled by a constant $\frac{s}{\lambda_{\max}}$, where we set s=0.8 to provide a ’stable’ matrix A; from this the indices $d_{Sym}\left(A\right)$ and $d_{Norm}\left(A\right)$ are calculated. Subsequently time series of length $T=10^{5}$ are generated according to Equation (32). The time series are reversed, and the matrix B is estimated using the *arfit* function and finally ${\overset{\leftarrow }{d}}_{Sym}\left(A\right)$ and ${\overset{\leftarrow }{d}}_{Norm}\left(A\right)$ is calculated. For the robustness of calculations, the whole process is repeated for 1000 different realizations of random matrix A. Two particular settings of the parameters are used to provide a comparison with subsequent analysis of complex real-world connectivity structures, namely n=42,D=15% and n=90,D=5%.

**Figure 2:**
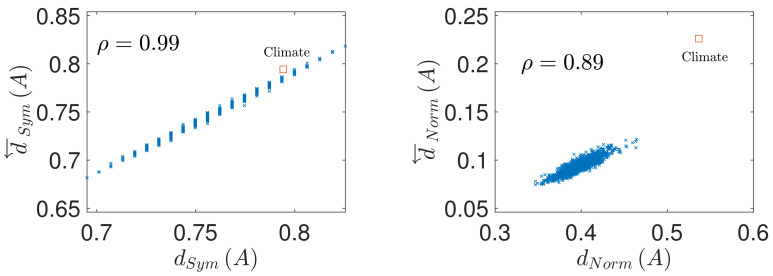
Prediction of the causal structure conservation and reversal from the coupling matrix properties, in a sample of random directed matrices with full diagonal (matrix size n=42, density of nonzero elements D=15%). (**Left**): causal structure conservation under time reversal ${\overset{\leftarrow }{d}}_{Sym}\left(A\right)$ as function of the coupling matrix symmetry $d_{Sym}\left(A\right)$. (**Right**): causal structure reversal under time reversal ${\overset{\leftarrow }{d}}_{Norm}\left(A\right)$ as function of the coupling matrix normality $d_{Norm}\left(A\right)$. ρ denotes the correlation coefficient between the considered quantities. The red square denotes the values for the climate model.

**Figure 3:**
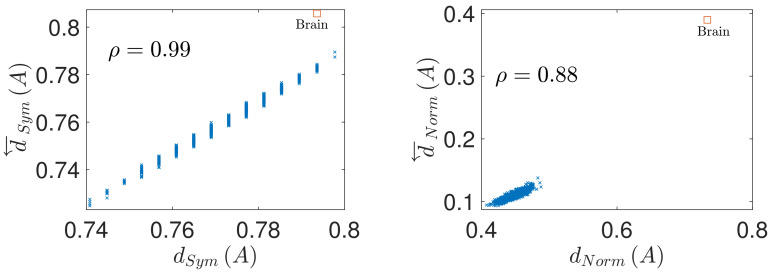
Prediction of the causal structure conservation and reversal from the coupling matrix properties, in a sample of random directed matrices with full diagonal (matrix size n=90, density of nonzero elements D=5%). (**Left**): causal structure conservation under time reversal ${\overset{\leftarrow }{d}}_{Sym}\left(A\right)$ as function of the coupling matrix symmetry $d_{Sym}\left(A\right)$. (**Right**): causal structure reversal under time reversal ${\overset{\leftarrow }{d}}_{Norm}\left(A\right)$ as function of the coupling matrix normality $d_{Norm}\left(A\right)$. ρ denotes the correlation coefficient between the considered quantities. The red square denotes the values for the brain model.

The results concerning the prediction of conservation of coupling matrix under time series reversal from symmetry of the input matrix A are shown in Figure 2 Left and Figure 3 Left. It is apparent, that the asymmetry of the coupling matrix $d_{Sym}\left(A\right)$ provides a very good prediction of the conservation of the coupling structure under time reversal ${\overset{\leftarrow }{d}}_{Sym}\left(A\right)$, with the respective Pearson correlation coefficient attaining the value of ρ=0.99 in both parameter settings. Similarly, Figure 2 Right and Figure 3 Right demonstrate that the deviation of the coupling matrix from normality quantified by $d_{Norm}\left(A\right)$ provides a very strong prediction of the coupling matrix reversal ${\overset{\leftarrow }{d}}_{Norm}\left(A\right)$, albeit weaker than in the case of the coupling conservation, with values ρ=0.89 and ρ=0.88 respectively.

### 3.3. Complex Connectivity Structures

So far, we showed that somewhat counter-intuitively, even in linear processes the time reversal has nontrivial effect on the coupling structure—in a general case it neither conserves nor reverses it, while these two special phenomena appear under quite specific conditions of normality (or more generally under fulfillment of the conditions given by Equation (10)) and symmetry (14), respectively. In the previous subsection, we demonstrated that indeed for random (ER) graphs, the coupling matrices of the time reversed process are relatively far from purely reversed or conserved causality, with the variability in the level of conservation/reversal being very well predicted by the symmetry/normality of the original (random) coupling matrix.

This motivates a question concerning the behaviour of more realistic models of real-world complex structures. To this end, we analyze the time-reversal effect on the coupling structure of linear vector autoregressive approximation of two archetypal examples of complex systems, the brain network and the climate network. We use the simple order 1 linear autoregressive process approximation of these processes, which is in line with the model order for which we derived that simplified condition for causal reversal/conservation. Therefore, the application of the corresponding indices of deviation from normality and symmetry is here well motivated. However, it should be kept in mind, that these model network examples, albeit realistically motivated, of course fall short of the complexity of the real brain and climate—both in terms of not capturing the nonlinear aspect of the brain/climate dynamics, and in simplifying the temporal dynamics by limiting the interactions to a single temporal lag. Indeed, even at the level of spatial and temporal sampling of the motivating datasets, the linear VAR(1) model is just an approximation, rather than an optimal model for the data—as model order criteria such as the Bayesian Information Criterion would suggest that a higher order VAR model would better describe the generating process. However, the quantification of the (deviation from) the causal reversal condition for higher-order VAR processes is not available in such a compact form; so we use only these order-one approximation of the real-world data coupling structures as motivating examples of somewhat more realistic connectivity matrices than provided by the ER random graph model.

#### 3.3.1. Earth’s Climate Network

The first example network is an approximation of a large-scale climate interaction network, constructed from regional daily temperature time series. The network has 42 nodes and was obtained by thresholding (binarizing) the interaction matrix in a data-fitted VAR(1) model to 15 percent density. In more detail, the original dataset contains the daily surface air temperature anomalies data obtained from the NECP/NCAR reanalysis dataset [14,15]. In particular, we used the daily air temperature fields at 1000 hPa level, spanning the period from 1 January 1948 to 31 December 2012 and sampled at $2.5^{\circ }$×$2.5^{\circ }$ angularly regular Gaussian grid. This original data contains more than 10,000 time series—a relatively dense grid covering the whole globe. The data were downsampled by remapping to a more suitable 42-point grid by applying an interpolation scheme based on the quasi-isotropic icosahedral grids [16] by a procedure implemented in the SCRIP package [17]. Details of the data origin and preprocessing are described in [18,19].

#### 3.3.2. Brain Network

The other network example is an approximation of brain interaction network that has 90 nodes and was obtained by thresholding (binarizing) the interaction matrix in a data-fitted VAR(1) model to 5% density. We use data obtained as part of a study on healthy subjects brain activity. The activity in 90 brain regions of 84 subjects were measured by functional magnetic resonance imaging, and temporally concatenated in order to provide sufficiently long time series (20,160 time points in total) for estimation of the coupling matrix. Details of the data origin and preprocessing are described in [18,19].

#### 3.3.3. Data Processing

As mentioned above, in preparing an approximate model of these real-world complex systems, we make the approximation that these datasets correspond to realizations of a VAR(1) process. Please note that albeit quite coarse and not capturing either the nonlinear aspects of the dynamics as well the plethora of time scales of these complex systems, the linear (autoregressive) approximation has been shown to reproduce sufficiently a range of properties of both the brain and climate network structure [20,21,22,23] and provide robust and interpretable estimate of causal interactions [22,24]. The estimated coupling matrix is then thresholded to predefined density (low values set to zero, only predefined amount of entries retained, in this case 15% for the climate matrix, 5% for the brain matrix), binarized (non-zero values set to 1) and subsequently normalized by the constant $\frac{s}{\lambda_{\max}}$ for the stationarity of the resulting process (all simulations were provided with setting s=0.8). This matrix defines the VAR(1) process from which we generate samples of arbitrary length *T* (in the simulations, we set $T=10^{5}$). The sample is then reverted and a VAR(1) model fitted to it using the *arfit* MATLAB function, providing thus an estimate of the matrix B. This matrix is then used to obtain an estimate of the values of ${\overset{\leftarrow }{d}}_{Norm}\left(A\right)$ and ${\overset{\leftarrow }{d}}_{Sym}\left(A\right)$.

#### 3.3.4. Analysis

The resulting values of deviation from symmetry and normality of the random as well as brain and climate coupling matrices, and imperfection of their coupling conservation/reversal upon time series reversal are shown in Figure 2 and Figure 3. How do the real-world matrices compare to the sets of matched random matrices? Clearly, both the brain and climate network show level of asymmetry relatively comparable to the directed random matrix of the same size and density. The level of coupling conservation upon time reversal is also similar to that of the asymmetric random matrices, suggesting that the approximate prediction of ${\overset{\leftarrow }{d}}_{Sym}\left(A\right)$ by ${\overset{\leftarrow }{d}}_{Sym}\left(A\right)$ works relatively well. To be more precise, both the climate and the brain matrix show slightly more asymmetry and less conservation under time reversal than random directed matrices of the same size and density, being safely above the 95-th percentile of the respective random matrix property distributions.

In contrast, in terms of normality, both the climate and brain coupling matrices show a pronounced deviation from normality, in particular substantially higher than directed random matrices of the same size and density. Notably, the deviation of the coupling matrix from perfect reversal under time reversal is also higher than for corresponding random matrices; as would be predicted by the high deviation of A from normality. These real-world complex system coupling matrices thus seem to substantially deviate from the intuition observed in two-dimensional linear systems, where the coupling tends to be approximately reversed upon time reversal; at least much more so that the realizations of randomly coupled linear networks do.

## 4. Discussion

The previous analysis has demonstrated, that even in very simple linear systems, the operation of time reversal has nontrivial effect on the coupling structure of the system, which only under very specific conditions corresponds to the reversal of the coupling structure, i.e., transposition of the matrix A defining the interactions in the case of the studied case of linear vector autoregressive process. For this particular case, we showed an explicit condition on the coupling matrix that needs to be fulfilled for the reversal to occur. Notably, this condition is far from fulfilled for a randomly coupled process, making perfect reversal an unlikely phenomenon; however we show that the deviation of the coupling matrix from normality, quantified by an intuitive index that we devised, seems to very well predict the level to which the reversal occurs. On the other hand, this index quantifying the deviation of the coupling matrix from normality is motivated by analytical derivation that showed normality of the coupling matrix as the key property for causal reversal for autoregressive processes of order one. For higher model orders, the condition for perfect reversal entails existence of solutions for a set of matrix polynomial Equation (4) that generally do not lend themselves to such an easy simplification as for order one that provided the normality condition; in particular, normality of all matrices $A_{1},\ldots ,A_{p}$ is not sufficient for perfect causal reversal in the VAR(p) case.

Examples of first order multivariate linear autoreggressive approximation of two real-world complex systems support the theoretical prediction of ubiquity of *imperfect reversal* of the coupling even for linear systems, and suggest that some if not many real-world systems, even when linear or linearly approximated, might be less prone to coupling reversal under time reversal than random coupled systems of the same size and density.

Of course, while perfect causal reversal seems reasonably predicted to be rare (as the coupling structure would have to fulfill very specific set of equations for it to occur), the level of (lack) causal reversal may vary widely depending on the structure of the connectivity. The analysis of example VAR(1) processes, including with random connectivity, real-world systems approximations as well as carefully constructed or simulation-derived counterexamples, only scratch the surface of the richness of real-world processes here; we only used examples from the VAR(1) family here as the theoretical analysis provides the convenient simplification to the normality condition. As noted previously, the VAR(1) approximation of the brain or climate data does in fact not correspond to the optimal model fit—the Bayesian Information Criterion would suggest to use a higher order model to described more accurately the full interaction structure of the data.

**Figure 4:**
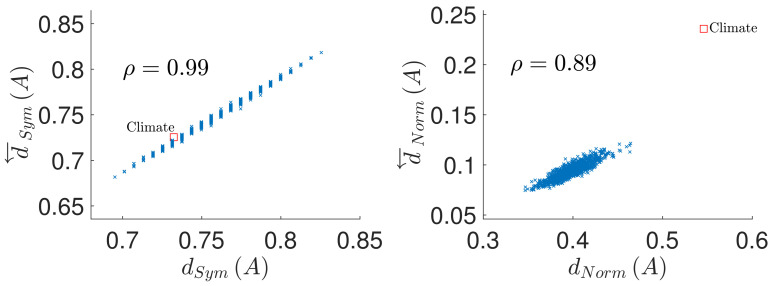
Prediction of the causal structure conservation and reversal from the coupling matrix properties, in a sample of random directed matrices with full diagonal (matrix size n=42, density of nonzero elements D=15%). (**Left**): causal structure conservation under time reversal ${\overset{\leftarrow }{d}}_{Sym}\left(A\right)$ as function of the coupling matrix symmetry $d_{Sym}\left(A\right)$. (**Right**): causal structure reversal under time reversal ${\overset{\leftarrow }{d}}_{Norm}\left(A\right)$ as function of the coupling matrix normality $d_{Norm}\left(A\right)$. ρ denotes the correlation coefficient between the considered quantities. The red square denotes the values for the VAR(1) model of the climate data downsampled by factor of 3 so that the model order p=1 provides the optimal model order according to the Bayesian Information Criterion.

**Figure 5:**
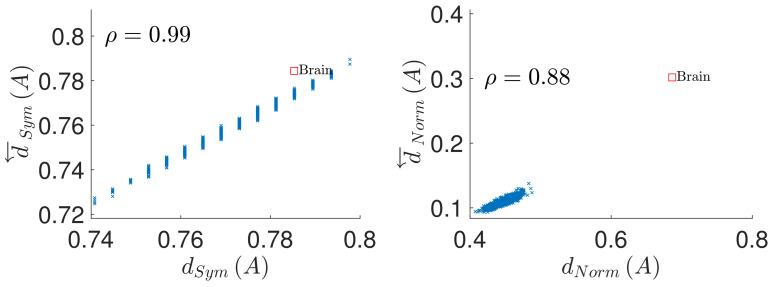
Prediction of the causal structure conservation and reversal from the coupling matrix properties, in a sample of random directed matrices with full diagonal (matrix size n=90, density of nonzero elements D=5%). (**Left**): causal structure conservation under time reversal ${\overset{\leftarrow }{d}}_{Sym}\left(A\right)$ as function of the coupling matrix symmetry $d_{Sym}\left(A\right)$. (**Right**): causal structure reversal under time reversal ${\overset{\leftarrow }{d}}_{Norm}\left(A\right)$ as function of the coupling matrix normality $d_{Norm}\left(A\right)$. ρ denotes the correlation coefficient between the considered quantities. The red square denotes the values for the VAR(1) model of the brain data downsampled by factor of 2 so that the model order p=1 provides the optimal model order according to the Bayesian Information Criterion.

Indeed, the VAR(1) approximation for fMRI data, albeit commonly used in practice, has been shown to be typically not optimal (although the correct model order widely varies depending on a range of properties of the fMRI datasets) [25]. Similarly, the climate temperature anomaly data are known to be only approximately linear and stationary (see [26]), and to contain nontrivial autocorrelation structure [27]. The fact that the VAR(1) is just an approximation—rather than the optimal model fit—for these datasets, however does not disprove the observation that even some more realistic rather than purely random connectivity patterns suggest that the deviation from causal reversal/conservation can be reasonably predicted from the deviation from the normality/symmetry. Indeed, qualitatively similar results are however obtained also on data downsampled by factor of 2 for the brain dataset and by factor of 3 for the climate dataset, for which indeed the Bayesian Information Criterion marks the order p=1 as the optimal model order (see Figure 4 and Figure 5 for the variant of the original Figure 2 and Figure 3 using the downsampled data). Indeed, it may be interesting to collate a catalogue of behaviour of different real-world systems in terms of causal reversal; however it would also entail the difficult task of differentiating properties of the original system and of the observation process and data quality and specific sampling biases—an enterprise beyond the scope of the current manuscript.

These results may seem counter-intuitive in light of some earlier reports concerning the coupling in time-reversed systems. The discussion of behaviour of causality measures on time-reversed observations has generally followed two different albeit interdependent motivations. First, it was (more or less explicitly) used to provide a control condition or construct a statistical test for causality detection [5,7,11]. Secondly, it is a subject of deeper discussion of the nature of causality and the meaning of the arrow of time in both linear and nonlinear natural systems [6].

In general, while the (approximate) reversal of causal interactions under time reversal has motivated both formulation of new causality estimation methods as well as theoretical discussion concerning fundamental properties of causality in linear vs. nonlinear systems, it is increasingly recognized that the issue is far from binary reversal/non-reversal. For application in real-world problems, the generalizability of the previous reports to networks of larger size would be of key importance. We hope that a more detailed analysis of the simplest VAR(1) approximation of such networks, albeit far from the real-world complexity, would thus help make next steps to extending the applicability of the proposed concepts to real-world contexts.

### 4.1. Relevance for Causality Indices Using Time-Reversal

The use of time-reversed time series for statistical evaluation of causal interactions in observed systems has been motivated by some perceived limitations of other available methods for causal inference. While Haaga et al. [7] motivates their interest in time series reversal by arguing that other methods including Granger causality, transfer entropy and methods such as convergent cross-mapping can suffer from biases and low statistical power, others such as Winkler et al. [5] and Haufe et al. [11] note that the application of Granger-causal measures combined with standard significance testing leads to the detection of spurious connectivity when applied to electrophysiology data that commonly suffer from source mixing and observational noise. Thus, while motivations vary, the use of time series reversal seems potentially relevant both in combination with nonlinear indices as well as with the widely used Granger causality analysis approach framed in the context of linear autoregressive processes [11].

Let us remind the reader that Granger causality analysis, named after Sir Clive Granger, who proposed this approach to time series analysis in a classical paper [4] follows the basic idea can be traced back to Wiener [28], stating that if the prediction of one time series can be improved by incorporating the knowledge of a second time series, then the latter can be said to have a causal influence on the former. This idea was formalised by Granger in the context of linear regression models. In the following, we outline the methods of assessment of Granger causality, following the description given in [29,30,31]. Consider two stochastic processes $X_{t}$ and $Y_{t}$ and assume they are jointly stationary. Let further the autoregressive representations of each process be:

(34) $$\begin{array}{c} X_{t}=\sum_{j=1}^{+\infty }a_{1j}X_{t-j}+\epsilon_{1t},\;\mathrm{var}\left(\epsilon_{1t}\right)=\Sigma_{1}, \end{array}$$

(35) $$\begin{array}{c} Y_{t}=\sum_{j=1}^{+\infty }d_{1j}Y_{t-j}+\eta_{1t},\;\mathrm{var}\left(\eta_{1t}\right)=\Gamma_{1}, \end{array}$$

and the joint autoregressive representation be:

(36) $$\begin{array}{cc} X_{t} & =\sum_{j=1}^{+\infty }a_{2j}X_{t-j}+\sum_{j=1}^{+\infty }b_{2j}Y_{t-j}+\epsilon_{2t},\; \end{array}$$

(37) $$\begin{array}{cc} Y_{t} & =\sum_{j=1}^{+\infty }c_{2j}X_{t-j}+\sum_{j=1}^{+\infty }d_{2j}Y_{t-j}+\eta_{2t},\; \end{array}$$

where the covariance matrix of the noise terms is:

(38) $$\begin{array}{c} \Sigma =\mathrm{Cov}\left(\begin{array}{c} \epsilon_{2t}\\ \eta_{2t} \end{array}\right)=\left(\begin{array}{cc} \Sigma_{2} & \Upsilon_{2}\\ \Upsilon_{2} & \Gamma_{2} \end{array}\right). \end{array}$$

The causal influence from *Y* to *X* is then quantified based on the decrease in the residual model variance when we include the past of *Y* in the model of *X*, i.e., when we move from the independent model given by Equation (34) to the joint model given by Equation (36):

(39) $$\begin{array}{c} F_{Y\to X}=\ln\frac{\Sigma_{1}}{\Sigma_{2}}. \end{array}$$

Similarly, the causal influence from *X* to *Y* is defined as:

(40) $$\begin{array}{c} F_{X\to Y}=\ln\frac{\Gamma_{1}}{\Gamma_{2}}. \end{array}$$

Clearly, the causal influence defined in this way is always nonnegative. When the system consists of more than two processes, one can use the above described pairwise Granger Causality Analysis to assess pairwise interactions in each direction. However, this approach suffers from inherent limitations. To give an example, a system consisting of three processes X,Y,Z, where *Z* drives both *X* and *Y*, but with different temporal lags, may erroneously show causal influence between *X* and *Y* even if these were not directly coupled. To deal with such situations, one can work with so-called conditional Granger Causality, that allows taking into account the variance explained by third variable(s). Please note that these definitions can be generalised to multivariate variables, as done e.g., in [32].

A key practical question is what can one infer from the value of the estimated Granger causality index, in particular when can one reject the hypothesis that there is no causal interaction between the two subsystems. In general, one can depend on some (asymptotically) known distribution of the test statistic (causal index), or construct an empirical distribution that would represent well the null hypothesis of no causal interaction; and compare the observed value against this empirical distribution. Additionally, as the value of the finite-sample estimate of the causality index might be affected by a bias depending on specific sample properties (e.g., length, autocorrelation or nonlinearity), various sophisticated heuristics have been proposed that would correct for this bias by subtracting the expected *baseline* value of the statistic.

In the context of Granger causality, Haufe et al. [11] suggested to contrast the Granger causality index $F_{X\to Y}$ with the one obtained from time-reversed time series ${\tilde{F}}_{\tilde{X}\to \tilde{Y}}$ and showed that this procedure, named time-reversed Granger causality (TRGC) robustly rejects causal interpretations on mixtures of independent signals. In 2016, this approach has been extended by Winkler et al. [5], who suggested three measures of causality based on the time reversal effect on the Granger causality index $F_{x\to y}$. Let us define the net Granger causality score as $F_{X\to Y}^{\left(\mathrm{net}\right)}=F_{X\to Y}-F_{Y\to X}$. One can then define the time-reversed net Granger causality score as

(41) $$\begin{array}{c} {\tilde{F}}_{\tilde{X}\to \tilde{Y}}^{\left(\mathrm{net}\right)}={\tilde{F}}_{\tilde{X}\to \tilde{Y}}-{\tilde{F}}_{\tilde{Y}\to \tilde{X}}, \end{array}$$

the difference-based time-reversed Granger causality score as

(42) $$\begin{array}{c} {\tilde{D}}_{X\to Y}=F_{X\to Y}-{\tilde{F}}_{\tilde{X}\to \tilde{Y}}, \end{array}$$

and the net difference-based time-reversed Granger causality score as

(43) $$\begin{array}{c} {\tilde{D}}_{X\to Y}^{\left(\mathrm{net}\right)}=F_{X\to Y}^{\left(\mathrm{net}\right)}-{\tilde{F}}_{\tilde{X}\to \tilde{Y}}^{\left(\mathrm{net}\right)}. \end{array}$$

Please note that in the final score ${\tilde{D}}_{X\to Y}^{\left(\mathrm{net}\right)}$, Winkler et al. [5] essentially combined two useful tricks for controlling for bias of estimates of causality—they contrast to the other direction of interaction (swapping source and target variable) *and* to the time-reversed quantity. These heuristics have been previously used separately in various contexts [33,34], also in conjunction with nonlinear data analysis approaches; see for instance the work of Haaga et al. [7] for the time-reversal-based causal inference using information-theoretical quantities, although their use of the time-reversed surrogate differs from the current construction.

As a key result, Winkler proposed and proved non-negativity of ${\tilde{D}}_{X\to Y}$ and ${\tilde{D}}_{X\to Y}^{\left(\mathrm{net}\right)}$ in bivariate system in which causality flows from variable *X* to *Y* but not in the other direction i.e., system represented by triangular matrix of size 2×2, i.e.,

(44) $$\begin{array}{c} {\tilde{D}}_{X\to Y}\geq 0 \end{array}$$

(45) $$\begin{array}{c} {\tilde{D}}_{X\to Y}^{\left(\mathrm{net}\right)}\geq 0. \end{array}$$

The authors have thus laid theoretical foundations to a more complex causal score, and in a range of numerical simulations showed its advantageous properties, particular in data confounded by observational (additive) noise, including scenarios where standard GC and other methods clearly fail, such as the presence of mixed autocorrelated noise. The (limitations) of applicability in nonlinear dynamical systems with a hidden influential variable [8] or with non-gaussian noise [9] were further studied.

Importantly, Winkler et al. [5] also state some open questions concerning the use of the net difference-based time-reversal Granger causality. In particular, its behaviour under bidirectional information flow and its extension to multivariate signals are considered to be important fields for further research. As we discuss below, a straightforward generalization of the appealing properties of coefficients ${\tilde{D}}_{X\to Y}$ and ${\tilde{D}}_{X\to Y}^{\left(\mathrm{net}\right)}$ in bidirectionally coupled or multivariate systems does unfortunately not hold in full generality; however we comment on conditions when it is applicable in its original form.

We envisage that in principle the knowledge of these conditions could either be used directly, in some form of pre-screening of these conditions as assumptions of the time-reversed Granger causality validity; or perhaps more efficiently, it may help develop a variant of the original time-reversed Granger causality approach that would be valid also for multivariate and bidirectionally coupled systems, providing thus a more generally applicable causal inference method.

### 4.2. Case of Normally Coupled Unidirectionally Systems

Importantly, even in the multivariate scenario, for unidirectionally coupled systems, we can state some positive results using the earlier findings concerning systems with a *normal* coupling matrix *A*. In particular, we can prove the same inequalities (44) for a multivariate system represented by VAR(1) process whose coupling matrix is normal and in which information flows from variable *X* to *Y* but not in the other direction i.e., $F_{X\to Y}>0$ but $F_{Y\to X}=0.$ As the coupling matrix is normal, the coupling in the time-reversed systems is perfectly reversed, that is $B=A^{⊤}$, and therefore $F_{X\to Y}={\tilde{F}}_{\tilde{Y}\to \tilde{X}}$. Then we have for the difference-based time-reversed Granger causality score in the causal direction:

(46) $$\begin{array}{c} {\tilde{D}}_{X\to Y}=F_{X\to Y}-{\tilde{F}}_{\tilde{X}\to \tilde{Y}}=F_{X\to Y}-F_{Y\to X}=F_{X\to Y}>0. \end{array}$$

In addition, similarly:

(47) $$\begin{array}{cc} {\tilde{D}}_{X\to Y}^{\left(\mathrm{net}\right)} & =F_{X\to Y}^{\left(\mathrm{net}\right)}-{\tilde{F}}_{\tilde{X}\to \tilde{Y}}^{\left(\mathrm{net}\right)}=F_{X\to Y}-F_{Y\to X}-\left({\tilde{F}}_{\tilde{X}\to \tilde{Y}}-{\tilde{F}}_{\tilde{Y}\to \tilde{X}}\right) \end{array}$$

(48) $$\begin{array}{cc} \; & =F_{X\to Y}-F_{Y\to X}-\left(F_{Y\to X}-F_{X\to Y}\right)=2F_{X\to Y}>0. \end{array}$$

Based on previous numerical simulations we can conjecture that the coefficient ${\tilde{D}}_{X\to Y}$ and ${\tilde{D}}_{X\to Y}^{\left(\mathrm{net}\right)}$ are also non-negative for systems whose representing matrix is “not far from normal” i.e., whose deviation from normality is close to zero. This would provide a promise that these causality scores introduced by Winkler et al. [5] could be reasonably applied for inference of causal direction in such family of multivariate systems.

### 4.3. Counterexample for Unidirectionally Coupled Systems

However, one can also construct examples, where these convenient properties fail, motivating more detailed study and development of related inference techniques. Already for a trivariate system represented by the VAR(1) process with a coupling matrix A and corresponding coupling matrix of reversed process B

(49) $$\begin{array}{c} A=\left(\begin{array}{ccc} 0.10 & 0.10 & 0.90\\ 0.00 & 0.70 & 0.90\\ 0.00 & 0.00 & 0.90 \end{array}\right),\;B=\left(\begin{array}{ccc} 0.20 & 0.35 & -0.00\\ -0.11 & 1.13 & -0.37\\ 0.33 & 0.08 & 0.37 \end{array}\right). \end{array}$$

with a corresponding Granger causality matrices

(50) $$\begin{array}{c} F=\left(\begin{array}{ccc} 0.01 & 0.07 & 0.79\\ 0.00 & 1.49 & 0.79\\ 0.00 & 0.00 & 0.79 \end{array}\right),\;\tilde{F}=\left(\begin{array}{ccc} 0.04 & 0.45 & 0.00\\ 0.01 & 1.64 & 0.09\\ 0.32 & 0.10 & 0.40 \end{array}\right) \end{array}$$

we obtain (using numerical simulations) matrices time-reversal Granger causality scores:

(51) $$\begin{array}{c} \tilde{D}=\left(\begin{array}{ccc} -0.02 & -0.38 & 0.79\\ -0.00 & -0.15 & 0.70\\ -0.32 & -0.10 & 0.39 \end{array}\right),\;{\tilde{D}}^{\left(\mathrm{net}\right)}=\left(\begin{array}{ccc} 0.00 & -0.37 & 1.11\\ 0.37 & 0.00 & 0.80\\ -1.23 & -0.80 & 0.00 \end{array}\right). \end{array}$$

As can be seen, there is a unidirectional causal effect from $X_{2}$ to $X_{1}$ (i.e., $F_{1,2}>0$, but $F_{2,1}=0$), but the time-reversed process has ’stronger’ causality in both directions, and thus the values of the difference-based time-reversed Granger causality scores between $X_{1}$ and $X_{2}$ are negative: ${\tilde{D}}_{X_{2}\to X_{1}}<0$. Moreover, even ${\tilde{D}}_{X_{2}\to X_{1}}^{\left(\mathrm{net}\right)}$ is negative; and thus inference depending solely on positivity of the scores would render wrong conclusion in this particular case.

### 4.4. Counterexample for Bidirectionally Coupled Systems

While for the unidirectionally coupled systems, Winkler et al. [5] proved analytically positivity of the proposed indices for the correct direction, the authors highlighted the interesting question of the behaviour of the time-reverted causality in the case of bidirectional causality as an open topic for further research. While we showed that in the general case the coupling matrix is not exactly reverted, for the application of the proposed causality scores it would be sufficient, if at least the dominant direction of causality was reverted—a behaviour that Paluš et al. [6] observed in example linear systems, in stark contrast to selected archetypal nonlinear systems in which causality indices typically conserved the dominant direction upon time reversal.

While we did not have a full analytical solution for the time-reversed coupling matrix and the resulting causality scores, we used here (similarly as in the three-dimensional unidirectionally coupled system) a systematic parameter search to test if any counterexamples can be numerically found. An example of such counterexample is shown below, already in dimension n=2. Please note that the dominant coupling in both forward and backward time-series is from $X_{2}$ to $X_{1}$, which is reflected both in the coupling matrices A and B as well as in the Granger causality matrices *F* and $\tilde{F}$, although the (net) difference-based time-reversed Granger causality matrices $\tilde{D},{\tilde{D}}^{\left(\mathrm{net}\right)}$ still detect the correct direction of causality (and thus in this respect, the lowest dimension of counterexample stays at three):

(52) $$\begin{array}{c} A=\left(\begin{array}{cc} 0.10 & 0.70\\ 0.50 & 0.60 \end{array}\right),\;B=\left(\begin{array}{cc} 0.17 & 0.64\\ 0.59 & 0.53 \end{array}\right) \end{array}$$

(53) $$\begin{array}{c} F=\left(\begin{array}{cc} 0.02 & 0.86\\ 0.36 & 0.70 \end{array}\right),\;\tilde{F}=\left(\begin{array}{cc} 0.04 & 0.67\\ 0.55 & 0.66 \end{array}\right) \end{array}$$

(54) $$\begin{array}{c} \tilde{D}=\left(\begin{array}{cc} -0.02 & 0.19\\ -0.19 & 0.03 \end{array}\right),\;{\tilde{D}}^{\left(\mathrm{net}\right)}=\left(\begin{array}{cc} 0.00 & 0.38\\ -0.38 & 0.00 \end{array}\right). \end{array}$$

In summary, while we have shown that a straightforward generalization of the useful concept of difference-based Granger causalities for causal inference in bidirectionally coupled or higher-dimensional systems is not available, the problem offers an interesting and rich set of open questions that may stimulate further development of this idea.

## 5. Conclusions

On top of the general results in the theoretical analysis section, the above discussed special cases demonstrated, that the situation concerning the coupling structure of the time-reversed systems is far from trivial already in the relatively simple case of linear autoregressive processes with white noise and a low dimension (*n* = 2 or *n* = 3). However, for some special cases (such as normality of the coupling matrix in the case of linear vector autoregressive model of order one), perfect causality reversal can be analytically shown, and for a broader family of high-dimensional random or complex structures, analytical tools seem to provide relevant clues concerning the time-reversed process coupling structure. We further illustrated that the coupling reversal or conservation does not follow strictly the dividing line between linear and nonlinear systems generally suggested by Paluš et al. [6], but deviate from it already within the realm of relatively simple linear systems; extending thus the problematic cases of linear systems with multiple delays and nonlinear systems in the synchronized regimes mentioned as possible exceptions to such rule of thumb in the original paper.

Notably, we used a theoretical framework that allows analytical derivation of conditions for causality reversal or conservation in linear systems. While this allowed us to derive the sufficient conditions for these phenomena in the simplest case of models with single time lag, further work may focus in particular on deriving necessary conditions, and generalizing the family of processes for which they are observed; although this may prove increasingly technical given the nature of the conditions that need to be considered.

## Data Availability

Example data presented in this paper are available upon request by email to the authors.

## Appendix A. Bivariate Case

In this section, we will derive the explicit form of reverse process of two special cases of VAR(1) process with white noise and coupling matrix $\left(\begin{array}{cc} a & b\\ 0 & d \end{array}\right).$

- d=0
  (A1) $$\begin{array}{c} A=-A_{1}=\left(\begin{array}{cc} a & b\\ 0 & 0 \end{array}\right) \end{array}$$
  The VAR(1) process with coupling matrix A is stationary if the equation $\det\left(A_{1}+\lambda I\right)=0$ has all the roots smaller than 1 in absolute value. Since
  (A2) $$\begin{array}{c} \det\left(A_{1}+\lambda I\right)=\det\left(\begin{array}{cc} -a+\lambda & -b\\ 0 & \lambda \end{array}\right)=\lambda \left(\lambda -a\right), \end{array}$$
  the process is stationary for |a|<1.
  We will show that reverse process is given by coupling matrix
  (A3) $$\begin{array}{c} B=-B_{0}^{-1}B_{1}=\left(\begin{array}{cc} a & 0\\ \frac{b\left(1-a^{2}\right)}{1+b^{2}} & 0 \end{array}\right) \end{array}$$
  and covariance matrix of the noise
  (A4) $$\begin{array}{c} B_{0}^{-1}{\left(B_{0}^{-1}\right)}^{⊤}=\left(\begin{array}{cc} 1+b^{2} & -ab\\ -ab & \frac{1+a^{2}b^{2}}{1+b^{2}} \end{array}\right). \end{array}$$
  Since $\det\left(B_{0}^{-1}{\left(B_{0}^{-1}\right)}^{⊤}\right)=1,$ hence $\det\left(B_{0}^{-1}\right)\neq 0$ and also $\det\left(B_{0}\right)\neq 0.$ Process is stationary because
  (A5) $$\begin{array}{c} \det\left(B_{1}+\lambda B_{0}\right)=\det\left(-B_{0}\left(\begin{array}{cc} a & 0\\ \frac{b\left(1-a^{2}\right)}{1+b^{2}} & 0 \end{array}\right)+\lambda B_{0}\right)=\det\left(B_{0}\right)\det\left(\begin{array}{cc} \lambda -a & 0\\ \frac{-b\left(1-a^{2}\right)}{1+b^{2}} & \lambda \end{array}\right)=0 \end{array}$$
  if λ=0∨λ=a and |a|<1 from the stationarity of the original process. The last step is to prove the fulfillment of the Equations (5) and (6). From (A4) follows
  (A6) $$\begin{array}{c} B_{0}^{⊤}B_{0}=\left(\begin{array}{cc} \frac{1+a^{2}b^{2}}{1+b^{2}} & ab\\ ab & 1+b^{2} \end{array}\right), \end{array}$$
  the Equation (5) is then satisfied because
  (A7) $$\begin{array}{cc} -A_{1}^{⊤} & =B_{0}^{⊤}B_{1}=-B_{0}^{⊤}B_{0}B_{0}^{-1}B_{1}=\left(\begin{array}{cc} \frac{1+a^{2}b^{2}}{1+b^{2}} & ab\\ ab & 1+b^{2} \end{array}\right)\left(\begin{array}{cc} a & 0\\ \frac{b\left(1-a^{2}\right)}{1+b^{2}} & 0 \end{array}\right)=\left(\begin{array}{cc} a & 0\\ b & 0 \end{array}\right) \end{array}$$
  and (6) is satisfied because
  (A8) $$\begin{array}{c} B_{1}^{⊤}B_{1}=B_{1}^{⊤}B_{0}B_{0}^{-1}B_{1}=A_{1}B_{0}^{-1}B_{1} \end{array}$$
  and
  (A9) $$\begin{array}{cc} \; & \left(\begin{array}{cc} 1+a^{2} & ab\\ ab & 1+b^{2} \end{array}\right)=I+A_{1}^{⊤}A_{1}=B_{0}^{⊤}B_{0}+B_{1}^{⊤}B_{1}=B_{0}^{⊤}B_{0}+A_{1}B_{0}^{-1}B_{1}=\\ \; & \left(\begin{array}{cc} \frac{1+a^{2}b^{2}}{1+b^{2}} & ab\\ ab & 1+b^{2} \end{array}\right)+\left(\begin{array}{cc} a & b\\ 0 & 0 \end{array}\right)\left(\begin{array}{cc} a & 0\\ \frac{b\left(1-a^{2}\right)}{1+b^{2}} & 0 \end{array}\right)=\left(\begin{array}{cc} 1+a^{2} & ab\\ ab & 1+b^{2} \end{array}\right). \end{array}$$
- a=0
  (A10) $$\begin{array}{c} A=-A_{1}^{⊤}=\left(\begin{array}{cc} 0 & b\\ 0 & d \end{array}\right) \end{array}$$
  The process is stationary if the equation $\det\left(A_{1}+\lambda I\right)=0$ has all the roots smaller than 1 in absolute value. Since
  (A11) $$\begin{array}{c} \det\left(A_{1}+\lambda I\right)=\det\left(\begin{array}{cc} \lambda & -b\\ 0 & -d+\lambda \end{array}\right)=\lambda \left(\lambda -d\right), \end{array}$$
  the process is stationary for |d|<1. We will show that reverse process is given by coupling matrix
  (A12) $$\begin{array}{c} B=-B_{0}^{-1}B_{1}=\frac{1}{1+b^{2}}\left(\begin{array}{cc} b^{2}d & bd^{2}\\ b & d \end{array}\right) \end{array}$$
  and covariance matrix of the noise
  (A13) $$\begin{array}{c} B_{0}^{-1}{\left(B_{0}^{-1}\right)}^{⊤}=\frac{1}{1+b^{2}}\left(\begin{array}{cc} {\left(1+b^{2}\right)}^{2}+b^{2}d^{2} & bd\\ bd & 1 \end{array}\right). \end{array}$$
  Similarly to previous case, since $\det\left(B_{0}^{-1}{\left(B_{0}^{-1}\right)}^{⊤}\right)=1,$ hence $\det\left(B_{0}^{-1}\right)\neq 0,$$\det\left(B_{0}\right)\neq 0$ and process is stationary because
  (A14) $$\begin{array}{c} \det\left(B_{1}+\lambda B_{0}\right)=\det\left(B_{0}\right)\det\left(\begin{array}{cc} \lambda -\frac{b^{2}d}{1+b^{2}} & \frac{-bd^{2}}{1+b^{2}}\\ \frac{-b}{1+b^{2}} & \lambda -\frac{d}{1+b^{2}} \end{array}\right)=\det\left(B_{0}\right)\left(\lambda -d\right)\lambda =0 \end{array}$$
  if λ=0∨λ=d and |d|<1 from the stationarity of the original process. The last step is to prove the fulfillment of the Equations (5) and (6). From (A14) follows
  (A15) $$\begin{array}{c} B_{0}^{⊤}B_{0}=\frac{1}{1+b^{2}}\left(\begin{array}{cc} 1 & -bd\\ -bd & {\left(1+b^{2}\right)}^{2}+b^{2}d^{2} \end{array}\right), \end{array}$$
  the Equation (5) is then satisfied because
  (A16) $$\begin{array}{c} -A_{1}^{⊤}=\frac{1}{{\left(1+b^{2}\right)}^{2}}\left(\begin{array}{cc} 1 & -bd\\ -bd & {\left(1+b^{2}\right)}^{2}+b^{2}d^{2} \end{array}\right)\left(\begin{array}{cc} b^{2}d & bd^{2}\\ b & d \end{array}\right)=\left(\begin{array}{cc} 0 & 0\\ b & d \end{array}\right) \end{array}$$
  and (6) is satisfied because
  (A17) $$\begin{array}{c} \left(\begin{array}{cc} 1 & 0\\ 0 & 1+b^{2}+d^{2} \end{array}\right)=I+A_{1}^{⊤}A_{1}=B_{0}^{⊤}B_{0}+B_{1}^{⊤}B_{1}=B_{0}^{⊤}B_{0}+A_{1}B_{0}^{-1}B_{1}=\\ \frac{1}{1+b^{2}}\left(\begin{array}{cc} 1 & -bd\\ -bd & {\left(1+b^{2}\right)}^{2}+b^{2}d^{2} \end{array}\right)+\frac{1}{{\left(1+b^{2}\right)}^{2}}\left(\begin{array}{cc} 0 & b\\ 0 & d \end{array}\right)\left(\begin{array}{cc} b^{2}d & bd^{2}\\ b & d \end{array}\right)=\left(\begin{array}{cc} 1 & 0\\ 0 & 1+b^{2}+d^{2} \end{array}\right). \end{array}$$
